# Supplementary material for: Modeling glioblastoma heterogeneity as a dynamic network of cell states
Source: Mol Syst Biol. 2021 Sep 16;17(9):e10105. doi: 10.15252/msb.202010105 (PMC8444284; doi:10.15252/msb.202010105)
Supplement: Supplementary file 5 — Source Data for Figure 3 [file MSB-17-e10105-s001.zip › Figure3A_sourcedata/GSEA_3065/hallmarks_state2.GseaPreranked.1623416269049/HALLMARK_TNFA_SIGNALING_VIA_NFKB.html]

Details for gene set HALLMARK\_TNFA\_SIGNALING\_VIA\_NFKB[GSEA]

|  || Dataset | state2 |
| Phenotype | NoPhenotypeAvailable |
| Upregulated in class | na\_neg |
| GeneSet | HALLMARK\_TNFA\_SIGNALING\_VIA\_NFKB |
| Enrichment Score (ES) | -0.49349675 |
| Normalized Enrichment Score (NES) | -1.9456875 |
| Nominal p-value | 0.0 |
| FDR q-value | 0.0 |
| FWER p-Value | 0.0 |
Table: GSEA Results Summary

  

Fig 1: Enrichment plot: HALLMARK\_TNFA\_SIGNALING\_VIA\_NFKB      
 Profile of the Running ES Score & Positions of GeneSet Members on the Rank Ordered List

  

| PROBE | GENE SYMBOL | GENE\_TITLE | RANK IN GENE LIST | RANK METRIC SCORE | RUNNING ES | CORE ENRICHMENT || 1 | BTG3 |  |  | 173 | 0.295 | 0.0162 | No |
| 2 | IER2 |  |  | 482 | 0.183 | 0.0048 | No |
| 3 | TUBB2A |  |  | 539 | 0.174 | 0.0193 | No |
| 4 | MARCKS |  |  | 630 | 0.159 | 0.0283 | No |
| 5 | CCND1 |  |  | 840 | 0.134 | 0.0217 | No |
| 6 | TGIF1 |  |  | 1150 | 0.108 | 0.0013 | No |
| 7 | EIF1 |  |  | 1261 | 0.101 | 0.0014 | No |
| 8 | IFNGR2 |  |  | 1538 | 0.085 | -0.0182 | No |
| 9 | LITAF |  |  | 1685 | 0.078 | -0.0247 | No |
| 10 | EGR1 |  |  | 1691 | 0.077 | -0.0161 | No |
| 11 | RELA |  |  | 1694 | 0.077 | -0.0073 | No |
| 12 | GADD45A |  |  | 1791 | 0.073 | -0.0090 | No |
| 13 | JUN |  |  | 1805 | 0.072 | -0.0019 | No |
| 14 | RCAN1 |  |  | 1903 | 0.068 | -0.0042 | No |
| 15 | RIPK2 |  |  | 1947 | 0.067 | -0.0010 | No |
| 16 | TNFAIP8 |  |  | 2308 | 0.053 | -0.0333 | No |
| 17 | YRDC |  |  | 2559 | 0.046 | -0.0547 | No |
| 18 | FOSL1 |  |  | 2584 | 0.045 | -0.0519 | No |
| 19 | RHOB |  |  | 2690 | 0.042 | -0.0583 | No |
| 20 | TNFAIP6 |  |  | 2720 | 0.041 | -0.0565 | No |
| 21 | SNN |  |  | 2846 | 0.038 | -0.0655 | No |
| 22 | SERPINB8 |  |  | 3093 | 0.032 | -0.0881 | No |
| 23 | DENND5A |  |  | 3135 | 0.031 | -0.0888 | No |
| 24 | SOD2 |  |  | 3366 | 0.026 | -0.1104 | No |
| 25 | REL |  |  | 3459 | 0.025 | -0.1173 | No |
| 26 | MAP2K3 |  |  | 3478 | 0.024 | -0.1164 | No |
| 27 | TNIP2 |  |  | 3698 | 0.020 | -0.1374 | No |
| 28 | MYC |  |  | 3842 | 0.018 | -0.1506 | No |
| 29 | JUNB |  |  | 3923 | 0.017 | -0.1572 | No |
| 30 | NFIL3 |  |  | 4213 | 0.012 | -0.1868 | No |
| 31 | NAMPT |  |  | 4573 | 0.006 | -0.2245 | No |
| 32 | SMAD3 |  |  | 4579 | 0.006 | -0.2244 | No |
| 33 | DUSP1 |  |  | 4653 | 0.005 | -0.2316 | No |
| 34 | VEGFA |  |  | 4721 | 0.004 | -0.2384 | No |
| 35 | NFE2L2 |  |  | 4821 | 0.002 | -0.2487 | No |
| 36 | ETS2 |  |  | 4918 | 0.001 | -0.2589 | No |
| 37 | ZBTB10 |  |  | 4934 | 0.001 | -0.2604 | No |
| 38 | TRIP10 |  |  | 4955 | 0.000 | -0.2625 | No |
| 39 | EHD1 |  |  | 5018 | -0.000 | -0.2691 | No |
| 40 | IER5 |  |  | 5041 | -0.001 | -0.2714 | No |
| 41 | DNAJB4 |  |  | 5172 | -0.003 | -0.2850 | No |
| 42 | TANK |  |  | 5234 | -0.004 | -0.2911 | No |
| 43 | NFKB1 |  |  | 5273 | -0.004 | -0.2946 | No |
| 44 | KLF10 |  |  | 5308 | -0.005 | -0.2977 | No |
| 45 | PANX1 |  |  | 5450 | -0.008 | -0.3119 | No |
| 46 | PDE4B |  |  | 5498 | -0.008 | -0.3159 | No |
| 47 | CEBPB |  |  | 5541 | -0.009 | -0.3194 | No |
| 48 | TNIP1 |  |  | 5561 | -0.009 | -0.3203 | No |
| 49 | DRAM1 |  |  | 5714 | -0.012 | -0.3352 | No |
| 50 | BIRC2 |  |  | 5833 | -0.014 | -0.3462 | No |
| 51 | HES1 |  |  | 6191 | -0.020 | -0.3822 | No |
| 52 | NFKBIA |  |  | 6199 | -0.020 | -0.3806 | No |
| 53 | NINJ1 |  |  | 6212 | -0.020 | -0.3795 | No |
| 54 | SOCS3 |  |  | 6224 | -0.020 | -0.3783 | No |
| 55 | DUSP4 |  |  | 6538 | -0.026 | -0.4088 | No |
| 56 | G0S2 |  |  | 6695 | -0.029 | -0.4220 | No |
| 57 | ATF3 |  |  | 6737 | -0.030 | -0.4229 | No |
| 58 | PHLDA1 |  |  | 6867 | -0.033 | -0.4328 | No |
| 59 | PLAUR |  |  | 6906 | -0.034 | -0.4328 | No |
| 60 | PPP1R15A |  |  | 7004 | -0.037 | -0.4389 | No |
| 61 | KLF6 |  |  | 7010 | -0.037 | -0.4351 | No |
| 62 | FOS |  |  | 7222 | -0.043 | -0.4527 | No |
| 63 | IRF1 |  |  | 7563 | -0.052 | -0.4830 | No |
| 64 | F3 |  |  | 7662 | -0.055 | -0.4870 | Yes |
| 65 | PDLIM5 |  |  | 7663 | -0.055 | -0.4806 | Yes |
| 66 | FOSL2 |  |  | 7698 | -0.056 | -0.4776 | Yes |
| 67 | BCL6 |  |  | 7753 | -0.058 | -0.4765 | Yes |
| 68 | B4GALT5 |  |  | 7780 | -0.059 | -0.4724 | Yes |
| 69 | PLPP3 |  |  | 7911 | -0.063 | -0.4789 | Yes |
| 70 | TAP1 |  |  | 7963 | -0.066 | -0.4766 | Yes |
| 71 | TIPARP |  |  | 8047 | -0.069 | -0.4774 | Yes |
| 72 | PLAU |  |  | 8131 | -0.073 | -0.4776 | Yes |
| 73 | BTG1 |  |  | 8156 | -0.075 | -0.4714 | Yes |
| 74 | SDC4 |  |  | 8172 | -0.076 | -0.4641 | Yes |
| 75 | MCL1 |  |  | 8200 | -0.077 | -0.4579 | Yes |
| 76 | KDM6B |  |  | 8266 | -0.081 | -0.4554 | Yes |
| 77 | F2RL1 |  |  | 8306 | -0.083 | -0.4498 | Yes |
| 78 | SLC2A3 |  |  | 8419 | -0.090 | -0.4511 | Yes |
| 79 | CCNL1 |  |  | 8435 | -0.091 | -0.4420 | Yes |
| 80 | SLC2A6 |  |  | 8451 | -0.093 | -0.4327 | Yes |
| 81 | CFLAR |  |  | 8485 | -0.095 | -0.4251 | Yes |
| 82 | CSF1 |  |  | 8560 | -0.101 | -0.4212 | Yes |
| 83 | ATP2B1 |  |  | 8584 | -0.102 | -0.4117 | Yes |
| 84 | ABCA1 |  |  | 8635 | -0.106 | -0.4047 | Yes |
| 85 | IRS2 |  |  | 8704 | -0.111 | -0.3988 | Yes |
| 86 | TSC22D1 |  |  | 8743 | -0.115 | -0.3893 | Yes |
| 87 | ID2 |  |  | 8781 | -0.120 | -0.3792 | Yes |
| 88 | SQSTM1 |  |  | 8790 | -0.121 | -0.3658 | Yes |
| 89 | PLK2 |  |  | 8803 | -0.123 | -0.3527 | Yes |
| 90 | BHLHE40 |  |  | 8804 | -0.123 | -0.3383 | Yes |
| 91 | PTPRE |  |  | 8861 | -0.131 | -0.3289 | Yes |
| 92 | NFAT5 |  |  | 8917 | -0.137 | -0.3186 | Yes |
| 93 | B4GALT1 |  |  | 8942 | -0.140 | -0.3047 | Yes |
| 94 | JAG1 |  |  | 8993 | -0.148 | -0.2927 | Yes |
| 95 | IL6ST |  |  | 9090 | -0.164 | -0.2837 | Yes |
| 96 | LDLR |  |  | 9117 | -0.170 | -0.2665 | Yes |
| 97 | CEBPD |  |  | 9235 | -0.205 | -0.2550 | Yes |
| 98 | BTG2 |  |  | 9239 | -0.207 | -0.2309 | Yes |
| 99 | PTX3 |  |  | 9249 | -0.212 | -0.2070 | Yes |
| 100 | SAT1 |  |  | 9279 | -0.224 | -0.1838 | Yes |
| 101 | FJX1 |  |  | 9282 | -0.225 | -0.1576 | Yes |
| 102 | PNRC1 |  |  | 9294 | -0.229 | -0.1319 | Yes |
| 103 | CDKN1A |  |  | 9301 | -0.230 | -0.1055 | Yes |
| 104 | PMEPA1 |  |  | 9313 | -0.241 | -0.0783 | Yes |
| 105 | TNC |  |  | 9403 | -0.383 | -0.0428 | Yes |
| 106 | CD44 |  |  | 9407 | -0.397 | 0.0035 | Yes |
Table: GSEA details [plain text format]

  

Fig 2: HALLMARK\_TNFA\_SIGNALING\_VIA\_NFKB: Random ES distribution      
 Gene set null distribution of ES for **HALLMARK\_TNFA\_SIGNALING\_VIA\_NFKB**
